# Silicon dioxide vaginal gel and short‐term high‐risk human papillomavirus DNA detectability: A randomized controlled trial

**DOI:** 10.1111/aogs.70332

**Published:** 2026-08-05

**Authors:** Julia M. Hecken, Clemens B. Tempfer, Katrin Klaus, Jan Scheich, Günther A. Rezniczek

**Affiliations:** ^1^ Department of Obstetrics and Gynecology, Marien Hospital Herne Klinikum der Ruhr‐Universität Bochum Herne Germany

**Keywords:** cervical cancer prevention, high‐risk HPV, HPV DNA detectability, human papillomavirus, randomized controlled trial, silicon dioxide, vaginal gel

## Abstract

**Introduction:**

Intravaginal products, including silicon dioxide‐based gels, are marketed to support short‐term high‐risk human papillomavirus (hrHPV) clearance or loss of detectability, but independent randomized evidence testing these short‐term claims against observation is limited. We evaluated whether daily intravaginal silicon dioxide‐based gel treatment increases the proportion of women with no detectable cervical hrHPV DNA compared with observation alone.

**Material and Methods:**

This prospective, single‐center, parallel‐group, randomized, open‐label controlled trial included women with PCR‐confirmed cervical hrHPV infection and no indication for immediate treatment. Participants were randomized 1:1 to 3 months of self‐administered daily intravaginal gel treatment or observation alone. The primary outcome was absence of detectable cervical hrHPV DNA by PCR at 3 months. Secondary outcomes during the randomized study period were patient‐reported satisfaction and adverse events. Trial registration: ClinicalTrials.gov, NCT05509413, https://clinicaltrials.gov/study/NCT05509413.

**Results:**

Between November 2022 and January 2025, 242 women were randomized, with 121 allocated to each group. Primary‐endpoint data were available for 208 women. At 3 months, hrHPV DNA was not detected in 18/99 women (18%) in the gel treatment group and 23/109 women (21%) in the observation group (absolute risk difference −2.9 percentage points, 95% CI −14 to 8; *p* = 0.60). A reduced post hoc adjusted sensitivity analysis did not materially change the interpretation of the primary comparison. Patient‐reported adverse events were more frequent in the gel treatment group than in the observation group (32/99 [32%] vs. 2/106 [2%]; absolute risk difference 30 percentage points, 95% CI 21 to 40; *p* < 0.001). Satisfaction scores were high in both groups but lower with gel treatment.

**Conclusions:**

In this randomized trial, 3 months of daily intravaginal silicon dioxide‐based gel treatment did not improve short‐term loss of detectable cervical hrHPV DNA compared with observation alone. The trial did not reproduce the large short‐term virologic benefit suggested by prior product‐related studies. The findings do not support routine use of this intervention solely to promote short‐term hrHPV non‐detectability in women without an indication for immediate treatment, particularly given the higher frequency of local adverse events.

## Graphical abstract

In an independent randomized trial of 242 women with PCR‐confirmed hrHPV infection, 3 months of silicon dioxide‐based vaginal gel did not improve hrHPV DNA non‐detectability compared with observation alone (18% vs. 21%). Patient‐reported adverse events were more frequent with gel (32% vs. 2%), and overall patient satisfaction was lower.

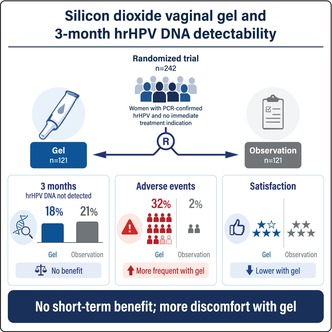

**Abbreviations**
CI: confidence interval
CIN: cervical intraepithelial neoplasia
HPV: human papillomavirus
hrHPV: high‐risk human papillomavirus
IQR: interquartile range

**Key message**

An independent randomized trial did not reproduce a short‐term benefit of silicon dioxide vaginal gel on three‐month PCR‐defined hrHPV non‐detectability compared with observation. Patient‐reported local adverse events were substantially more frequent with gel use.

## 1 INTRODUCTION

Persistent infection with high‐risk human papillomavirus (hrHPV) is a central causal factor in the development of cervical cancer and its precursors.^1^, ^2^ Most hrHPV infections become clinically undetectable over time, and low‐grade cervical abnormalities frequently regress, but loss of HPV DNA detectability on a clinical assay should not be equated with proven viral eradication.^2^, ^3^, ^4^, ^5^ Nevertheless, short‐term changes in HPV test results are often used in studies and product claims evaluating interventions intended to influence the natural course of hrHPV infection.

In routine clinical practice, women with hrHPV infection, normal cytology, or low‐grade cytological abnormalities are generally managed by observation unless immediate excisional or ablative treatment is indicated. Despite this, topical vaginal products are marketed and used to support hrHPV clearance, loss of HPV detectability, or regression of low‐grade cervical findings.^6^ One such product is a vaginal gel containing silicon dioxide, citric acid, and selenium.^7^ The proposed mechanism of action includes adsorption of viral particles and modulation of the local microenvironment, although direct antiviral effects have not been demonstrated.^8^, ^9^

Product‐related observational studies and one industry‐sponsored randomized trial have reported short‐term improvements in cytological surrogate markers, such as p16/Ki‐67 expression, regression of low‐grade squamous intraepithelial lesions, or reductions in hrHPV prevalence following use of this gel.^8^, ^10^ These studies, which also informed the original sample‐size assumptions of the present trial, suggested a large short‐term effect over a three‐month treatment period. However, they were limited by small sample size, heterogeneous inclusion criteria, variable outcome definitions, and reliance on surrogate or composite outcomes.^9^, ^10^

These prior data and product‐related claims create a specific evidence question: whether the reported short‐term benefit is reproducible in an independent randomized comparison against observation, accounting for spontaneous loss of hrHPV detectability. Such a comparison is clinically relevant because women without an indication for immediate treatment may be offered topical products despite the absence of guideline‐supported pharmacological treatment for hrHPV infection itself.

The objective of this randomized controlled trial was to evaluate whether a three‐month course of daily intravaginal silicon dioxide‐based gel treatment increases the proportion of women with no detectable cervical hrHPV DNA by PCR at the end of treatment compared with observation alone. Secondary objectives included assessment of patient‐reported satisfaction and treatment‐related adverse events during the randomized study period. Outcomes after the three‐month randomized comparison were assessed descriptively because crossover to gel treatment was offered thereafter.

## 2 MATERIAL AND METHODS

### 2.1 Study design and setting

This was a prospective, single‐center, parallel‐group, randomized, open‐label, controlled interventional trial conducted at the Department of Obstetrics and Gynecology, Marien Hospital Herne, Ruhr‐Universität Bochum, Germany. The confirmatory part of the trial compared 3 months of daily intravaginal silicon dioxide‐based gel treatment with observation alone for the primary endpoint of absence of detectable cervical hrHPV DNA by PCR at 3 months. Analyses after the three‐month primary endpoint were exploratory because women in the observation group with persistent detectable hrHPV could cross over to gel treatment thereafter.

The study protocol was approved by the local institutional ethics committee (reference 22‐7635‐BR) and was prospectively registered at ClinicalTrials.gov (NCT05509413) before enrollment of the first participant. All participants provided written informed consent.

### 2.2 Participants

Eligible participants were women with PCR‐confirmed cervical hrHPV infection and either normal cytology or low‐grade cytological abnormalities, including atypical squamous cells of undetermined significance (ASC‐US), atypical glandular cells of undetermined significance (AGUS), or low‐grade squamous intraepithelial lesions (LSIL), classified according to the Munich Nomenclature III.^11^ All participants underwent baseline colposcopy. Directed cervical biopsies were obtained according to colposcopic and clinical assessment, and histological findings were used to exclude disease requiring immediate treatment. The extent of baseline colposcopic and histological assessment is summarized in Table S1.

Women were eligible if no immediate excisional or ablative treatment was indicated after colposcopic and histological assessment. In one case, histology showed cervical intraepithelial neoplasia grade 2, corresponding to HSIL according to Bethesda terminology. Because conservative management was considered acceptable according to local clinical guidance, this finding was discussed with the participant, who chose continued study participation.

Exclusion criteria included pregnancy, immunosuppression, active oncological disease or immunotherapy, relevant hematological disorders, and major language barriers precluding informed consent or study participation.

### 2.3 Randomization and masking

Participants were randomized in a 1:1 ratio to either intravaginal silicon dioxide‐based gel treatment or observation alone. Randomization was based on a computer‐generated allocation list using permuted blocks of variable size (four and six).^12^ The allocation list and the sequentially numbered, opaque, sealed envelopes containing the assignments were prepared before study initiation by a member of the study team who was not involved in patient recruitment, enrolment, or treatment. Personnel recruiting, enrolling, or treating participants had no access to the allocation sequence. Envelopes were opened only after written informed consent had been obtained.

The trial was open‐label because the intended comparator was observation alone, reflecting the clinical management alternative for women without an indication for immediate treatment. A placebo gel would have enabled blinding and improved interpretation of subjective outcomes, particularly satisfaction and patient‐reported local adverse events, but the primary study question was pragmatic: whether adding the marketed gel to observation improves short‐term hrHPV non‐detectability compared with observation alone. The primary endpoint was laboratory‐defined, and hrHPV testing was performed without knowledge of treatment allocation. Subjective outcomes were interpreted in light of the open‐label design.

### 2.4 Intervention and control

Participants allocated to the intervention group were instructed to self‐administer 5 mL of the silicon dioxide‐based vaginal gel DeflaGyn® once daily for 3 months. According to the product information, the gel contains silicon dioxide (10 mg), citric acid (24.8 mg), and selenium (0.25 mg) per 5 mL application. Participants allocated to the control group received no active treatment and were managed with observation alone. After completion of the three‐month primary‐endpoint assessment, participants in the observation group with persistent detectable hrHPV were offered crossover to gel treatment.

### 2.5 Outcomes

The primary outcome was absence of detectable cervical hrHPV DNA by PCR at 3 months. This endpoint was defined operationally as a negative hrHPV PCR result at the three‐month follow‐up visit and was not interpreted as proof of biological viral eradication.

Secondary outcomes during the randomized 0‐ to 3‐month study period were patient‐reported satisfaction and patient‐reported adverse events. Satisfaction was assessed using a pragmatic 11‐point numerical rating scale from 0 to 10, with higher values indicating greater satisfaction; the scale was not a disease‐specific validated instrument. Adverse events and treatment‐related complaints were assessed using participant questionnaires completed either electronically or on paper, according to participant preference. Reported adverse events were extracted from free‐text responses and categorized descriptively before analysis. No standardized toxicity grading scale was applied.

hrHPV non‐detectability after the post‐randomization observation period was assessed descriptively as an exploratory outcome. Registered outcomes and their corresponding reporting locations are summarized in Table S5.

### 2.6 Patient and public involvement

Patients and members of the public were not involved in the design, conduct, reporting, or dissemination planning of this study. Patient‐reported satisfaction and adverse events were included to capture participant experience during the randomized study period.

### 2.7 Sample size

The original sample‐size calculation was performed using G*Power 3.1.9.2 for a two‐sided *z* test comparing two independent proportions.^13^ The calculation was designed to test the large short‐term benefit suggested by earlier product‐related studies evaluating silicon dioxide‐based gel treatment in women with abnormal cytology and/or low‐grade cervical lesions.^10^ It assumed a three‐month hrHPV non‐detectability rate of 15% in the observation group and 40% in the intervention group, corresponding to a 25‐percentage‐point absolute increase. With *α* = 0.05, 95% power, and equal allocation between groups, 80 participants per group were required. Allowing for an anticipated dropout rate of 5%, the original target sample size was 168 participants, with 84 participants per group.

After the original recruitment target had been reached and three‐month primary‐endpoint data were available for this cohort, the primary endpoint was analyzed as originally planned. This analysis showed that the hrHPV DNA non‐detectability proportions and treatment effect assumed for the original sample‐size calculation, which had been derived from earlier product‐related studies, were not consistent with the observed data in our study population. No confirmatory conclusion was drawn from this initial analysis. Further exploratory analyses were stopped, and recruitment was extended in order to increase the precision of the treatment‐effect estimate and to improve the ability of the trial to detect a smaller treatment effect than originally assumed. The final sample included 242 randomized participants, with 121 participants in each group. Descriptively, increasing the randomized sample size from 84 to 121 participants per group increased nominal power from approximately 96% to more than 99% under the original 15% versus 40% assumptions, and from approximately 65% to approximately 81% for a smaller 15‐percentage‐point absolute difference, illustrated as 15% versus 30%. The extension of recruitment and these recalculations were not part of a prespecified adaptive sample‐size re‐estimation design.

### 2.8 HPV testing and data collection

Cervical samples for hrHPV testing were collected at baseline and at the three‐month follow‐up visit using the Rovers® Cervex‐Brush® (Rovers Medical Devices B.V., Oss, the Netherlands). Additional cervical samples were collected during the post‐randomization observation period when applicable. DNA extraction was performed using the QIAamp® DNA Mini Kit (Qiagen, Hilden, Germany), and hrHPV detection was performed using the GeneProof Human Papillomavirus Screening PCR Kit (GeneProof a.s., Brno, Czech Republic), following the manufacturers' protocols. The hrHPV PCR assay detects a broad panel of hrHPV genotypes and differentiates HPV types 16, 18, and 45. Laboratory personnel performing hrHPV testing were unaware of treatment allocation.

Study data were collected and managed using REDCap (Research Electronic Data Capture).^14^

### 2.9 Statistical analyses

The primary analysis was performed in the evaluable primary‐endpoint population, defined as all randomized participants with evaluable hrHPV PCR results at the three‐month follow‐up visit. The primary outcome was compared between randomized groups using the *χ* ^2^ test. The treatment effect was reported as the absolute risk difference with 95% confidence interval (CI), calculated using the Newcombe method based on Wilson score intervals.^15^ Because some randomized participants did not have evaluable primary‐endpoint data, the primary analysis was accompanied by sensitivity analyses for missing outcome data.

Continuous variables were summarized as medians with interquartile ranges and compared using non‐parametric tests. Categorical variables were summarized as counts and percentages and compared using *χ* ^2^ tests. For satisfaction scores, between‐group differences were analyzed using a non‐parametric test. For adverse events, the proportion of participants reporting at least one adverse event was compared between groups during the randomized 0‐ to 3‐month study period.

Missing primary outcome data were addressed in sensitivity analyses using extreme‐case assumptions. In the best‐case scenario for the intervention group, all participants with missing primary outcome data in the intervention group were assumed to have no detectable hrHPV DNA and all participants with missing primary outcome data in the observation group were assumed to have detectable hrHPV DNA. The worst‐case scenario used the opposite assumptions. A post hoc adjusted sensitivity analysis of the primary endpoint was performed using logistic regression. Because the number of hrHPV DNA non‐detectability events was limited, this model was restricted to treatment group and selected baseline variables and was interpreted as exploratory.

Analyses after the three‐month primary endpoint were descriptive and exploratory because treatment exposure was no longer determined by randomized allocation after crossover. No formal adjustment for multiplicity was applied; secondary and exploratory analyses were interpreted accordingly.

All statistical tests were two‐sided, and *p* < 0.05 was considered statistically significant. Data preparation was performed in Microsoft Excel, and statistical analyses were conducted using SigmaPlot 16 (Grafiti LLC, Palo Alto, CA) and R.^16^

## 3 RESULTS

### 3.1 Participant flow and baseline characteristics

**FIGURE 1:**
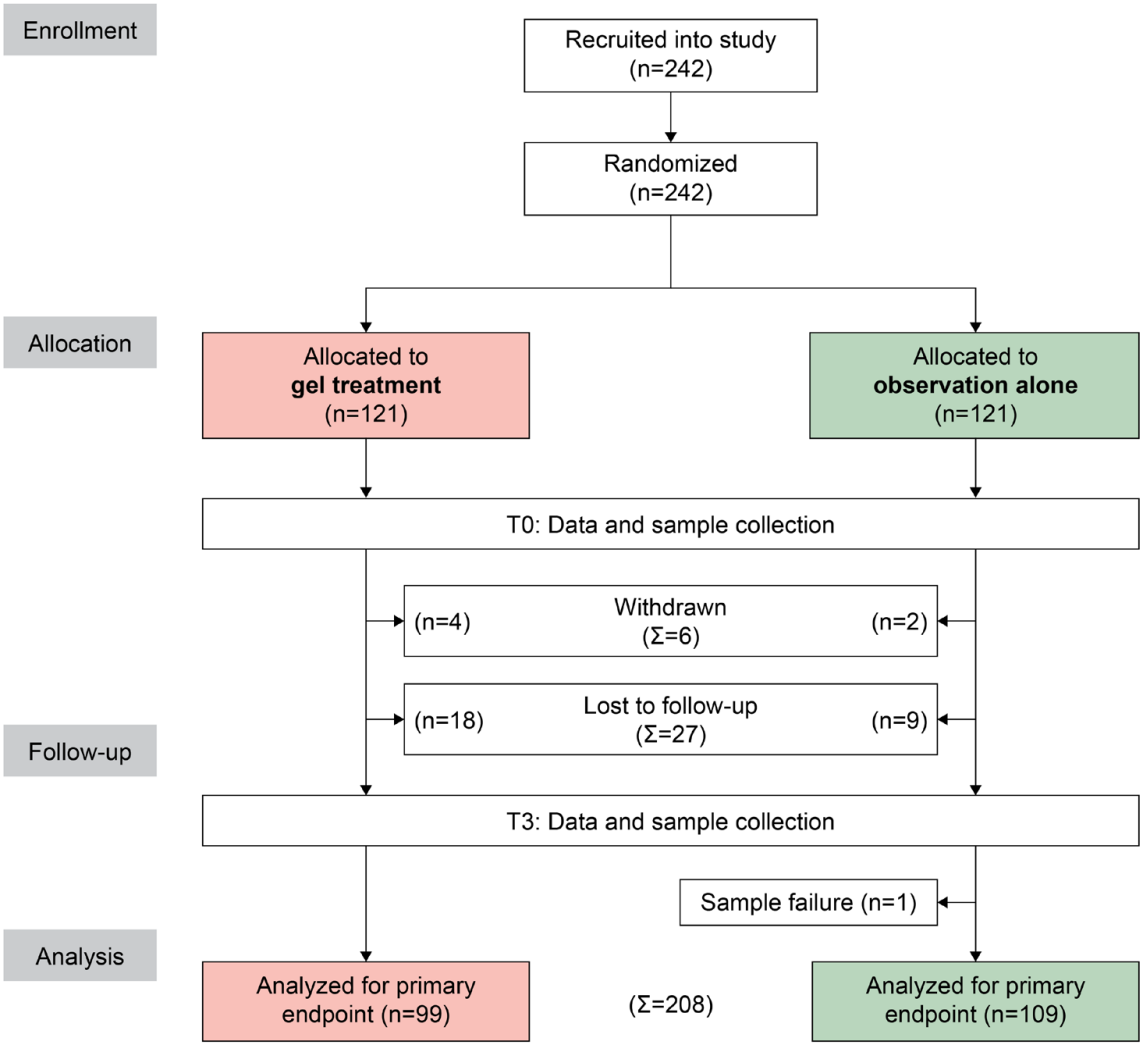
Flow diagram of the randomized controlled trial. T0, baseline examination; T3, three‐month follow‐up.

Between November 2022 and January 2025, 242 women were randomized, with 121 allocated to gel treatment and 121 to observation alone (Figure 1). Primary‐endpoint data at 3 months were available for 208 women. Missing primary‐endpoint data were more frequent in the gel treatment group than in the observation group. Reasons for missing primary‐endpoint data are shown in Figure 1.

Baseline demographic, clinical, cytological, colposcopic, and histopathological characteristics were broadly comparable between groups. All randomized women had confirmed hrHPV infection at baseline. The distribution of HPV genotypes, including HPV 16 and/or 18, was similar between groups (Table 1). Baseline histology showed CIN1/LSIL in 3 women in the gel treatment group and 2 women in the observation group, and HSIL/CIN2 in 1 woman in the observation group (Table S1).

**TABLE 1:** Patient characteristics, cytology, and HPV status.

	Gel treatment	Observation only
Number of patients	121	121
Patient characteristic		
Age, years	48 (38–56)	49 (41–58)
Body mass index, kg/m^2^	24.4 (21.8–28.5)	24.7 (22.1–29.7), *N* = 120
Parity	1 (0–2), *N* = 114	1 (0–2), *N* = 120
Currently pregnant	0/121 (0%)	0/121 (0%)
Allergies, any	48/120 (40%)	59/121 (49%)
To a compound of the gel	0/121 (0%)	0/121 (0%)
Tobacco use		
Currently smoking	29/120 (24%)	35/121 (29%)
Ever smoked	54/120 (45%)	52/121 (43%)
Never smoked	66/120 (55%)	69/121 (57%)
Alcohol consumption, any	61/121 (50%)	66/120 (55%)
Drug abuse, any	1/121 (0.8%)	0/120 (0%)
Concomitant disease, any	66/121 (55%)	74/121 (61%)
Prescription drug use, any	68/121 (56%)	81/121 (67%)
Immunomodulating conditions, any	3/121 (2.5%)	7/121 (5.8%)
Referring cytology		
NILM (PAP I, II‐a)	71/121 (59%)	76/121 (63%)
ASC‐US (PAP II‐p)	17/121 (14%)	16/121 (13%)
AGC endocervical NOS (PAP II‐g)	1/121 (0.8%)	0/121 (0%)
ASC‐H (PAP III‐p)	7/121 (5.8%)	4/121 (3.3%)
AGC endocervical favor neoplastic (PAP III‐g)	1/121 (0.8%)	2/121 (1.7%)
LSIL (PAP IIID1)	17/121 (14%)	15/121 (12%)
HSIL (PAP IIID2)	7/121 (5.8%)	8/121 (6.6%)
High‐risk HPV status		
Positive	121/121 (100%)	121/121 (100%)
Positive for 16 and/or 18	72/117 (62%)	63/119 (53%)

*Note*: Values are *n*/*N* (%) or medians (interquartile ranges).

Abbreviation: HPV, human papillomavirus.

### 3.2 Primary and secondary outcomes at the three‐month randomized endpoint

Primary and secondary outcomes during the randomized three‐month study period are shown in Table 2. At 3 months, hrHPV DNA non‐detectability did not differ between the gel treatment and observation groups. Extreme‐case sensitivity analyses for missing primary outcome data were dependent on the assumptions applied and did not support a robust treatment effect in either direction. A reduced post hoc adjusted sensitivity analysis including randomized group, baseline HPV 16 and/or 18 status, and age did not materially change the interpretation of the primary comparison (Table S2).

**TABLE 2:** Primary and secondary outcomes at the three‐month randomized endpoint.

Outcome	Gel treatment	Observation only	Effect estimate	*p*
Primary outcome				
hrHPV DNA not detected at 3 months	18/99 (18%)	23/109 (21%)	RR 0.86 (95% CI 0.50 to 1.5); risk difference −2.9 percentage points (95% CI −14 to 8.0)	0.60
Secondary outcomes				
Patient‐reported adverse event/complication, any	32/99 (32%)	2/106 (2%)	RR 17 (95% CI 4.2 to 70); risk difference 30 percentage points (95% CI 21 to 40)	<0.001
Satisfaction score, 0–10 numerical rating scale	10 (8–10), *N* = 98	10 (9–10), *N* = 105	Cliff's δ −0.21, favoring observation	0.003

*Note*: Values are *n*/*N* (%) or median (interquartile range). Relative risks compare gel treatment with observation only. Satisfaction was assessed using an 11‐point numerical rating scale from 0 to 10, with higher values indicating greater satisfaction, and Cliff's δ was used to compare the probability that a randomly selected participant in the gel group had a higher score than a randomly selected participant in the observation group with the reverse probability; negative values indicate lower satisfaction in the gel group.

Abbreviations: CI, confidence interval; hrHPV, high‐risk human papillomavirus; RR, relative risk.

Patient‐reported satisfaction was high in both groups but showed a distributional shift toward lower scores in the gel treatment group (Table 2). Patient‐reported adverse events or complications were more frequent in the gel treatment group than in the observation group (Table 2). The most frequently reported adverse events were burning sensations, vaginal bleeding, and itching (Table 3).

**TABLE 3:** Patient‐reported adverse events and treatment‐related complaints.

	Gel treatment	Observation only	*p*
0–3 months study period			
Number of patients	99	109	
Patients reporting adverse events*	32/99 (32%)	2/106 (1.9%)	<0.001
Burning sensations	17	0	
Bleeding	11	0	
Itching	3	0	
Abdominal pain	3	0	
Dryness	2	0	
Inflammation	1	1	
Other	3	1	
3–6 months study period	(crossed over to gel treatment)	(discontinued gel treatment or did not cross over)	
Number of patients	68	74	
Patients reporting adverse events*	24/66 (36%)	3/71 (4.2%)	<0.001
Burning sensations	13	2	
Bleeding	8	0	
Abdominal pain	4	2	
Itching	1	0	
Dryness	1	0	
Other	3	0	
Further complaints of patients while under gel treatment (independent of study period)			
Number of patients	167		
Patients voicing complaints*	54/165 (33%)		
Gel too liquid/flows out even after prolonged time	47		
Unpleasant smell of the product	19		

*Note*: Values are *n*/*N* (%) or counts. Denominators for adverse‐event and complaint outcomes reflect available questionnaire data. *p* Values: *χ* ^2^ test; for the 3‐ to 6‐month period, *p* values are descriptive and compare adverse‐event reporting by post‐randomization treatment exposure, not by randomized allocation.

* Multiple adverse events or complaints per patient could be reported, therefore category counts may not add up to the number of patients.

### 3.3 Exploratory post‐randomization and registered outcomes

Participant flow during the post‐randomization observation period is shown in Figure S1. After the three‐month primary‐endpoint assessment, 79 women in the observation group with persistent detectable hrHPV DNA crossed over to gel treatment, whereas seven continued observation. Among women with persistent detectable hrHPV DNA after initial gel treatment, gel use was discontinued.

Across the full observation period of up to 6 months, hrHPV DNA was not detected in 44/110 women (40%) initially allocated to observation alone and 26/100 women (26%) initially allocated to gel treatment (Table S3). Among women with persistent detectable hrHPV DNA at 3 months, hrHPV DNA was no longer detected between 3 and 6 months in 20/68 women (29%) who crossed over from observation to gel treatment and 8/70 women (11%) who discontinued gel treatment after initial gel use (Table S4). These findings were considered descriptive because treatment exposure after 3 months was no longer determined by randomized allocation.

Six‐month satisfaction remained high and did not differ by original randomized group or by actual treatment exposure during the post‐randomization observation period (Table S5). No progression was observed among participants with dysplasia at baseline, and no newly diagnosed dysplasia or malignancy was observed during follow‐up. Among five participants with baseline CIN1/LSIL, four showed resolution on later biopsy and one had persistent CIN1 at 1 year. One participant with baseline HSIL/CIN2 remained hrHPV‐positive and eventually underwent conization, with no dysplasia or malignancy detected in the excised tissue. Exact six‐month histological assessment was not available because the 6‐month visit included cervical sampling for hrHPV PCR but no biopsy. Registered outcomes and their corresponding analyses are summarized in Table S5.

## 4 DISCUSSION

In this prospective randomized trial designed to test the short‐term benefit suggested by product‐related studies and claims, 3 months of daily intravaginal silicon dioxide‐based gel treatment did not increase the proportion of women with no detectable cervical hrHPV DNA by PCR compared with observation alone. The observed point estimate did not favor gel treatment, and the upper limit of the 95% confidence interval was well below the 25‐percentage‐point absolute increase assumed in the original sample‐size calculation. The trial therefore did not reproduce the large short‐term virologic effect suggested by earlier studies. Smaller effects, delayed effects, or subgroup‐specific effects, however, cannot be excluded.

These findings are important because prior studies of silicon dioxide‐based gel treatment reported improvements in cytological surrogate markers, regression of low‐grade cervical lesions, or reductions in hrHPV prevalence over short follow‐up intervals.^8^, ^9^, ^10^ Such changes are difficult to interpret without randomized comparison because spontaneous loss of hrHPV detectability and regression of low‐grade abnormalities are common.^2^, ^3^, ^4^ The observation arm in the present trial directly accounts for this background natural history. The present endpoint should be understood as PCR‐defined loss of detectable hrHPV DNA, not as proof of biological viral eradication.^5^

Gel treatment was associated with substantially more patient‐reported local adverse events during the randomized study period. These events mainly included burning sensations, vaginal bleeding, and itching. Patient‐reported satisfaction was high in both groups but lower among women allocated to gel treatment. Because the trial was open‐label and did not include a placebo gel, subjective outcomes such as satisfaction and adverse‐event reporting may have been influenced by awareness of group allocation or by the daily intravaginal application procedure itself. Nevertheless, these outcomes reflect the treatment burden experienced by participants under pragmatic use conditions.

The main strengths of the study are its prospective randomized design, independent conduct without industry sponsorship, clinically relevant comparison with observation alone, and laboratory‐defined primary endpoint. The trial was designed to test whether the short‐term effect suggested by prior product‐related evidence could be reproduced beyond the spontaneous loss of hrHPV detectability expected under observation. Baseline colposcopic and histological assessment reduced the risk that disease requiring immediate treatment influenced management.

Several limitations should be considered. The study was single‐center, which may limit generalizability. The open‐label design was chosen because observation alone represented the intended pragmatic comparator but lack of blinding remains relevant for subjective outcomes. Primary‐endpoint data were missing for some randomized participants, particularly in the gel treatment group, and this reduces certainty despite sensitivity analyses. The original sample‐size calculation was based on a large expected treatment effect derived from earlier small product‐related studies, and the subsequent recruitment extension was not part of a prespecified adaptive design; the trial was therefore not designed to exclude small differences in hrHPV non‐detectability. The endpoint was assessed at 3 months and does not establish durable HPV control, histological regression, or prevention of CIN progression. A trial designed to assess treatment of HSIL/CIN2+ or histologically proven regression would require a different population, endpoint, and management framework. In addition, treatment crossover after the three‐month primary endpoint precluded causal interpretation of later outcomes. Findings from the post‐randomization observation period were therefore descriptive and hypothesis‐generating only.

Overall, the trial does not provide evidence that silicon dioxide‐based gel treatment improves short‐term PCR‐defined hrHPV non‐detectability during the randomized three‐month treatment period. The findings argue against a short‐term benefit of the magnitude reported or implied by earlier product‐related evidence. Given the increased frequency of local adverse events and treatment‐related complaints, the balance between potential short‐term virologic benefit and treatment burden appears unfavorable for routine use of this intervention solely to promote hrHPV non‐detectability.

## 5 CONCLUSION

In this randomized controlled trial, 3 months of daily intravaginal silicon dioxide‐based gel treatment did not improve short‐term loss of detectable cervical hrHPV DNA compared with observation alone. The findings do not support routine use of this intervention solely to promote short‐term hrHPV non‐detectability in women without an indication for immediate treatment, particularly given the higher frequency of local adverse events. Future studies should use blinded, placebo‐controlled designs and longer‐term histological outcomes if smaller, delayed, or disease‐regression effects are to be evaluated.

## AUTHOR CONTRIBUTIONS

Julia M. Hecken, Clemens B. Tempfer, and Günther A. Rezniczek conceptualized the study. Julia M. Hecken, Katrin Klaus, and Jan Scheich contributed to investigation and data curation. Julia M. Hecken, Katrin Klaus, and Günther A. Rezniczek performed the formal analyses. Clemens B. Tempfer and Günther A. Rezniczek contributed to methodology, supervision, and study resources. Julia M. Hecken and Günther A. Rezniczek validated the data and analyses. Günther A. Rezniczek prepared the visualizations. Julia M. Hecken, Clemens B. Tempfer, and Günther A. Rezniczek drafted the original manuscript. All authors contributed to review and editing of the manuscript, approved the final version, and accept responsibility for the integrity of the work.

## FUNDING INFORMATION

This study was funded exclusively through internal institutional resources of the Department of Obstetrics and Gynecology, Marien Hospital Herne, Ruhr‐Universität Bochum, Germany. No external financial or non‐financial support was received. The funder had no role in study design; data collection, analysis, or interpretation; manuscript preparation; or the decision to submit the manuscript for publication.

## CONFLICT OF INTEREST STATEMENT

The authors declare no conflicts of interest.

## ETHICS STATEMENT

The study was approved by the Ethics Committee of the Faculty of Medicine, Ruhr‐Universität Bochum, Germany (reference 22‐7635‐BR; approval date: 2022‐10‐12). The trial was prospectively registered at ClinicalTrials.gov (NCT05509413; registration date: 2022‐08‐18; URL: https://clinicaltrials.gov/study/NCT05509413) before enrollment of the first participant on 2022‐11‐08. All participants provided written informed consent before study participation.

## Supporting information


Figure S1.
**Tables S1–S5**.

## Data Availability

The datasets generated and analyzed during the current study are not publicly available because of patient confidentiality and institutional data‐protection requirements. Deidentified data may be made available from the corresponding author upon reasonable request, subject to ethical approval, data‐protection requirements, and applicable data‐use agreements.
