# Supplementary material for: Silicon dioxide vaginal gel and short‐term high‐risk human papillomavirus DNA detectability: A randomized controlled trial
Source: Acta Obstet Gynecol Scand. 2026 Aug 5;105(11):2256–64. doi: 10.1111/aogs.70332 (PMC13443988; doi:10.1111/aogs.70332)
Supplement: Supplementary file 1 [file AOGS-105-2256-s001.docx]

Silicon dioxide vaginal gel and short-term high-risk human papillomavirus DNA detectability: a randomized controlled trial

Julia M. Hecken, MD^1^, Clemens B. Tempfer, MD^1^, Katrin Klaus^1^, Jan Scheich^1^, and Günther A. Rezniczek, PhD^1^

^1^ Department of Obstetrics and Gynecology, Marien Hospital Herne, Klinikum der Ruhr-Universität Bochum, Herne, Germany

Supporting Information

**Supporting Information Table S1.** Colposcopy and histopathology findings

**Supporting Information Table S2.** Reduced adjusted sensitivity analysis of three-month hrHPV DNA non-detectability

**Supporting Information Table S3.** Cumulative hrHPV DNA non-detectability by six months, by initial randomized group

**Supporting Information Table S4.** hrHPV DNA non-detectability between three and six months among women with persistent hrHPV infection at three months

**Supporting Information Table S5.** Registered outcomes and location of reporting

**Supporting Information Figure S1.** Participant flow during the post-randomization observation period.

**Supporting Information Table S1.** Colposcopy and histopathology findings

|  | **Gel treatment** | **Observation only** |
| --- | --- | --- |
| Number of patients | 121 | 121 |
| e taken | 4 (3–4) | 4 (4–4) |
| *Colposcopic findings (RIO classification)* |  |  |
| Normal | 112 (93) | 113 (93) |
| Minor changes | 4 (3.3) | 7 (5.8) |
| Major changes | 1 (0.8) | 0 |
| Other | 4 (3.3) | 1 (0.8) |
| *Type of transformation zone* |  |  |
| Type 1 | 30 (25) | 37 (31) |
| Type 2 | 18 (15) | 17 (14) |
| Type 3 | 68 (56) | 66 (55) |
| Not visible | 5 (4.1) | 1 (0.8) |
| *Histological results* |  | [1]^1^ |
| Negative for dysplasia | 118 (98) | 117 (98) |
| LSIL | 3 (2.5) | 2 (1.7) |
| HSIL | 0 | 1 (0.8) |

Values are counts (percentage proportions) or medians (interquartile ranges). Numbers in square brackets indicate the number of missing values. LSIL, low-grade squamous intraepithelial lesion; HSIL, high-grade squamous intraepithelial lesion.

^1^ No sample was taken during colposcopy.

**Supporting Information Table S2.** Reduced adjusted sensitivity analysis of three-month hrHPV DNA non-detectability

| **Variable** | **Adjusted odds ratio** | **95% CI** | ***P*** |
| --- | --- | --- | --- |
| Gel treatment vs observation alone | 0.82 | 0.41 to 1.6 | 0.58 |
| HPV 16 and/or 18 vs other hrHPV | 0.83 | 0.41 to 1.7 | 0.60 |
| Age, per 10-year increase | 0.87 | 0.65 to 1.2 | 0.37 |

Logistic regression model including randomized group, baseline HPV 16 and/or 18 status, and age. The dependent variable was absence of detectable hrHPV DNA at 3 months. The analysis included 208 randomized women with evaluable 3-month hrHPV PCR results. This post hoc model was performed as an adjusted sensitivity analysis and was interpreted as exploratory. Model fit: likelihood ratio χ² = 1.54, p = 0.67; Hosmer–Lemeshow χ² = 13.4, p = 0.10.

**Supporting Information Table S3.** Cumulative hrHPV DNA non-detectability by six months, by initial randomized group

| **Initial randomization group** | **hrHPV DNA not detected (%) [95% CI]** | **Detectable hrHPV DNA (%)** | **Total** |
| --- | --- | --- | --- |
| Observation only | 44 (40) [31–49] | 66 (60) | 110 |
| Gel treatment | 26 (26) [18–35] | 74 (74) | 100 |
| Total | 70 (33) | 140 (67) | 210 |

Values are counts (percentage proportions; within rows) and [95% confidence intervals (CI) calculated using the Wilson method].

**Supporting Information Table S4.** hrHPV DNA non-detectability between three and six months among women with detectable hrHPV DNA at three months

| **Post-randomization subgroup** | **hrHPV DNA not detected (%) [95% CI]** | **Detectable hrHPV DNA (%)** | **Total** |
| --- | --- | --- | --- |
| Observation only → Gel treatment | 20 (29) [19–42] | 48 (71) | 68 |
| Observation only (entire 6 months) | 1 (25) [4.6–70] | 3 (75) | 4 |
| Gel treatment → Stop | 8 (11) [5.9–21] | 62 (89) | 70 |
| Total | 29 (20) | 113 (80) | 142 |

Values are counts (percentage proportions; within rows) and [95% confidence intervals (CI) calculated using the Newcombe/Wilson method].

**Supporting Information Table S5.** Registered outcomes and location of reporting

| **Registered outcome** | **Time frame** | **Reported in** | **Result / comment** |
| --- | --- | --- | --- |
| hrHPV-positive cervical smears /  hrHPV detectability | 3 and/or 6 months | Table 2; Supporting Information Table S3 | Three-month result is the randomized primary endpoint. Six-month result is descriptive because of crossover. |
| Rate of progression among participants with dysplasia at baseline | 3 and 6 months | Supporting Information Table S5 | No progression was observed. Of 2 baseline CIN1 cases in the observation group, both resolved on follow-up biopsy after 8 and 9 months. Of 3 baseline CIN1 cases in the gel group, 2 resolved after 7.5 and 8 months and 1 persisted at 1 year, followed by LLETZ. The one participant in the observation group who had baseline HSIL/CIN2, remained hrHPV-positive, and eventually underwent conization; no dysplasia or malignancy was detected in the excised tissue. Exact 6-month histology was not available because the 6-month visit included hrHPV PCR sampling but no biopsy. |
| Rate of newly diagnosed dysplasia or malignancy, not present at baseline | 3 and 6 months | Supporting Information Table S5 | No newly diagnosed dysplasia or malignancy was observed or reported during the follow-up. Exact 6-month histological assessment was not available because the 6-month visit included cervical sampling for hrHPV PCR but no biopsy. |
| Complications | 0–3 months | Table 2; Table 3 | Table 2 gives patient-level outcome;  Table 3 gives event categories. |
| Patients’ satisfaction | 3 and 6 months | Three-month randomized outcome: Table 2. Six-month descriptive outcome: Supporting Information Table S5. | Three-month satisfaction is reported as a randomized-period secondary outcome. Six-month satisfaction did not differ by original randomized group: median 10 (IQR 8–10), N=86, in the gel group versus 10 (8–10), N=89, in the observation group; p=0.75. Six-month satisfaction also did not differ by actual treatment exposure during the 3–6 month period: median 10 (8–10), N=66, with gel treatment versus 10 (8–10), N=72, with observation/no gel; p=0.41. Six-month analyses are descriptive because treatment exposure after the three-month primary endpoint was affected by crossover. |

**Supporting Information Figure S1.** Participant flow during the post-randomization observation period.
